# Supplementary material for: Exploration of key markers driving ferroptosis in the progression of non-alcoholic fatty liver disease
Source: PLoS One. 2025 Nov 7;20(11):e0320399. doi: 10.1371/journal.pone.0320399 (PMC12594402; doi:10.1371/journal.pone.0320399)
Supplement: Attachment — Submitted filename: Review of Article-20250303.docx [file pone.0320399.s001.docx]

**Summary:**

In this study, the author used bioinformatics analysis to identify 1,770 ferroptosis-related differentially expressed genes (DEGs) in NAFLD patients compared with healthy controls. In addition, functional pathway analysis revealed notable changes in metabolic and DNA repair pathways, accompanied by shifts in 19 immune cell types, including increased infiltration of 12 in NAFLD. A PPI network identified 41 key genes, with 25 showing high diagnostic accuracy (AUC > 0.85). These findings illuminate the molecular underpinnings of NAFLD and propose novel genetic targets for early diagnosis and personalized therapy. However, there are still some comments that need to be addressed by the author.

**Comments:**

1. Please revise the manuscript’s layout to ensure consistent spacing, as some sections need additional space while others have too much spacing between words.
2. ”In recent years, the research of mechanisms of NAFLD has increased. They found that dysregulation of iron metabolism is closely related to the progression of NAFLD.“ This sentence could be improved as “In recent years, research on the mechanisms of NAFLD has grown substantially, and multiple studies have shown that dysregulation of iron metabolism is closely associated with its progression.”
3. I can easily find numerous papers discussing ferroptosis biomarkers in the context of NAFLD induction. Could the author please introduce the latest findings in this field?
4. Insufficient Clinical and Demographic Details: There is no mention of patient demographics (e.g., age, sex, BMI) or clinical parameters that could influence NAFLD progression and confounding factors (like comorbidities or medication use) are not accounted for, making it difficult to interpret how these might affect the results.
5. Lack of Tissue-Specific or Sample-Type Clarity: It is not explicitly stated whether the GEO datasets are based on liver biopsy tissues, blood samples, or another source. NAFLD-related biomarkers can vary significantly based on the sample type (e.g., circulating biomarkers versus liver-tissue-specific markers).
6. No Mention of Randomization or Blinding: As is typical with bioinformatics studies using publicly available data, there’s no clear statement about how patient samples were selected or whether there was any randomization or blinding in the original studies. This can limit control over potential selection bias.
