# Supplementary material for: Exploration of key markers driving ferroptosis in the progression of non-alcoholic fatty liver disease
Source: PLoS One. 2025 Nov 7;20(11):e0320399. doi: 10.1371/journal.pone.0320399 (PMC12594402; doi:10.1371/journal.pone.0320399)
Supplement: Attachment — Submitted filename: Rebuttal Letter.docx [file pone.0320399.s002.docx]

Dear Editors,

Thank you so much for taking the time to review our manuscript. We greatly appreciate your and the reviewer’s comments and suggestions on our initial manuscript, which helps us to improve the overall quality of the paper. We have responded to each question you and the reviewer have raised in the following content. We hope these modifications would meet the requirements. If there’s any other further changes required, please let us know. Thank you very much.

**Editor’s comments:**

1: The new terminology for NAFLD is metabolic dysfunction associated liver disease (MASLD). Is changing the term will change your results? and are patients have diabetes mellitus or not?

Our study subjects and analysis procedure are based on NAFLD cases labeled by public database (GEO). The collection and categorization of these data are based on internationally-recognized diagnostic criteria for NAFLD at that time. MASLD, as a new term proposed in recent years, highly overlaps with NAFLD in their core diagnostic standard, which both emphasizes liver fat deposition in the context of metabolic dysfunction. Therefore, changing the term will not affect our gene expression analysis, grouping strategy, and main conclusions. As for whether patients have diabetes, due to limited completeness of patient information in public databases, our study was not able to obtain detailed metabolic comorbidities of our samples.

2: Typing errors require correction e.g., in introduction, ofmechanisms, accuracyand........etc.

Thank you for pointing those out. We have re-checked the grammar and formatting issues and corrected them to ensure the accuracy in expression.

3:Introduction: Add a paragraph about the role ferroptosis in NAFLD or MASLD because the introduction is with few references.

We added a description on the role of ferroptosis in the pathogenesis and progression of NAFLD or MASLD in the introduction, as well as several latest and authoritative review articles, such as PMID: 33495651, 36828775, 33268902, to enhance the completeness and theoretical basis of the research background.

4: The sample size of control in GSE130970 dataset is small. It can be mentioned as a limitation of the study.

We used multiple datasets merging and batch effect correction to minimize the impact of insufficient sample size. We have added an explanation in the "Discussion" section that the small sample size of control in GSE130970 may have an impact on the representativeness and statistical power of the analysis results, and described it as one of the limitations of this study. Please see page19.

5. It is a little be confused about the correlation test, is it Pearson or Spearman?

We mainly used Spearman correlation coefficient to evaluate the correlation between variables, especially when analyzing the relationship between immune cell infiltration and key gene expression. For correlation analysis between WGCNA module feature genes and traits, Pearman was also used due to its linear relationship based on the expression matrix.

6. The area under the curve needs to be supplemented with probability and 95% confidence interval

We supplemented all ROC analysis curve with AUC probability values and 95% confidence interval for each gene, and provided detailed explanations in the "Diagnostic Value of Hub Genes" section and corresponding legends and texts.

7. Improving the quality of figures is important to be more attractive.

We fully understand the importance of high-quality figures in enhancing the overall expression of the manuscript. Considering all the figures in the study have high resolution, complete information, and meet the journal format in their scientific visualization, we think currently all the figures can display the core research content clearly and accurately. Because the figures are designed with scientificity and readability to the greatest extent, we think there is no need for further modification.

**Reviewer’s comments:**

1. Please revise the manuscript’s layout to ensure consistent spacing, as some sections need additional space while others have too much spacing between words.

We have adjusted paragraph spacing and layout to maintain consistency.

2. ”In recent years, the research of mechanisms of NAFLD has increased. They found that dysregulation of iron metabolism is closely related to the progression of NAFLD.“ This sentence could be improved as “In recent years, research on the mechanisms of NAFLD has grown substantially, and multiple studies have shown that dysregulation of iron metabolism is closely associated with its progression.”

We have replaced the sentence accordingly.

3. I can easily find numerous papers discussing ferroptosis biomarkers in the context of NAFLD induction. Could the author please introduce the latest findings in this field?

We have noticed the increasing research on ferroptosis and its function as a biomarker of NAFLD in recent years. Multiple studies have systematically summarized the main mechanisms, key molecules, and the prospect of clinical application. The innovation of this study lies in the use of systematic bioinformatics method, combined with multiple databases and algorithms, to screen for the key gene of novel ferroptosis that has the potential of early diagnostic value and to explore their immune mechanisms in depth. Therefore, only brief citation and explanation were provided in the introduction and discussion. If further expansion of literature review is needed, relevant content can be supplemented in the subsequent research or articles.

4. Insufficient Clinical and Demographic Details: There is no mention of patient demographics (e.g., age, sex, BMI) or clinical parameters that could influence NAFLD progression and confounding factors (like comorbidities or medication use) are not accounted for, making it difficult to interpret how these might affect the results.

Due to the use of public database (GEO) in this study, detailed demographic and clinical parameters such as age, gender, BMI, comorbidities, and medica use were not disclosed in the original data, therefore we were not able to include those data into our analysis. Our research mainly focuses on high-throughput bioinformatics exploring of large sample in public data, which aims to screen for potential molecular markers and mechanisms.

5. Lack of Tissue-Specific or Sample-Type Clarity: It is not explicitly stated whether the GEO datasets are based on liver biopsy tissues, blood samples, or another source. NAFLD-related biomarkers can vary significantly based on the sample type (e.g., circulating biomarkers versus liver-tissue-specific markers).

Our GEO databases used (GSE130970 and GSE89632) are based on liver tissue samples, which are highly correlated with the tissue specificity of the pathogenesis of NAFLD. We have added relevant information in the data and method section, as well as in the abstract and discussion section.

6. No Mention of Randomization or Blinding: As is typical with bioinformatics studies using publicly available data, there’s no clear statement about how patient samples were selected or whether there was any randomization or blinding in the original studies. This can limit control over potential selection bias.

The data used in this study is from public database (GEO). The collection, grouping, and management of relevant data were completed by their original research team according to their respective study designs. Our team strictly followed the data usage policy during analysis process and did not make any subjective adjustments or re-assignments to the sample. Therefore, we are not able to further intervene with randomization or blinding of the original data collection process. In response to this, we added this as a limitation to the study.
